# Direct Experience While Eating in a Sample With Eating Disorders and Obesity

**DOI:** 10.3389/fpsyg.2018.01373

**Published:** 2018-09-07

**Authors:** Joaquim Soler, Ausiàs Cebolla, Matilde Elices, Daniel Campos, Ginés Llorca, David Martínez-Rubio, Cristina Martínez-Brotóns, Mercedes Jorquera, Xavier Allirot, Cristina Carmona, Verónica Guillen, Cristina Botella, Rosa M. Baños

**Affiliations:** ^1^Department of Psychiatry, Research Institute of the Sant Pau Hospital, Autonomous University of Barcelona, Barcelona, Spain; ^2^Centro de Investigación Biomédica en Red de Salud Mental, Madrid, Spain; ^3^Department of Personality, Assessment and Psychological Treatments, University of Valencia, Valencia, Spain; ^4^CIBERObn Ciber Physiopathology of Obesity and Nutrition, Madrid, Spain; ^5^Mental Health Research Group, Hospital del Mar Medical Research Institute, Barcelona, Spain; ^6^Department of Basic Psychology, Psychobiology and Clinical Psychology, Universitat Jaume I, Castellón de la Plana, Spain; ^7^Instituto de Investigación Sanitaria Aragón, Zaragoza, Spain; ^8^Consorci Hospitalari Provincial de Castelló, Castellón de la Plana, Spain; ^9^Catholic University of Valencia “San Vicente Mrtir”, Valencia, Spain; ^10^Clinical Center PREVI, Valencia, Spain; ^11^Basque Culinary Center, Donostia, Spain

**Keywords:** eating disorders, obesity, anorexia nervosa, bulimia nervosa, mindful eating, mindfulness, direct experience

## Abstract

**Background:** Individuals with eating disorders might be characterized by lower levels of direct engagement with the eating experience. This study aims to explore similarities and differences in direct experience while eating in four different weight conditions and healthy controls (HCs): anorexia nervosa (AN), bulimia nervosa (BN), eating disorders not otherwise specified (EDNOS), and obesity (OB).

**Methods**: A total sample of 143 women were recruited. Participants were asked to eat an orange slice and write down 10 things about the experience of eating, classifying the focus of these thoughts as either experiential (“direct experience”) or analytical (“thinking about”). A direct experience index (DEI) was calculated by dividing the number of times a participant classified an experience as a “direct experience” (the numerator) by the total number of observations. Participants completed the Five Facet Mindfulness Questionnaire (FFMQ) and rated their level of anxiety after the task.

**Results**: Between-groups significant differences were found on the DEI, with individuals in the OB group scoring higher than AN and BN, and similar to HC. After the task, the AN group reported significantly higher anxiety levels than HC, and EDNOS reported more anxiety than HC and OB. Between-group significant differences were also found for all the FFMQ facets. **Conclusion**: AN and BN presented lower access to direct experience while eating. Individuals with OB did not respond in the same way as the other clinical groups, showing a similar performance to HC.

## Introduction

Eating disorders (EDs) and obesity (OB) share maladaptive eating behaviors, such as restraint or emotional eating (Konttinen et al., 2009; Baños et al., 2014). Among the EDs, anorexia nervosa (AN) is characterized by restricting food intake to pursue thinness, and bulimia nervosa (BN) is characterized by binging and purging (American Psychiatric Association [APA], 2013).

Mindfulness refers to a way of self-regulating attention to focus it on the present moment experience with an attitude of curiosity, openness, and acceptance of bodily sensations, thoughts, and emotions (Bishop et al., 2004). Deficits in dispositional mindfulness have been reported in ED (Lavender et al., 2011; Cowdrey and Park, 2012; Elices et al., 2017) and OB (Camilleri et al., 2015), compared to healthy populations. When mindfulness refers to the process of eating, it is called mindful eating; Framson et al. (2009) described it as “non-judgmental awareness of physical and emotional sensations associated with eating.” Mindful eating correlates inversely with the severity of eating disorders (EDs) (Soler et al., 2013) and seems to increase positive mood (Meier et al., 2017) and healthy food choices (Allirot et al., 2017). Clinical trials of mindfulness-based interventions in samples with ED or OB showed beneficial effects, including: weight reduction (e.g., O’Reilly et al., 2014; Olson and Emery, 2015; Carrière et al., 2018), ED and depressive symptom improvement (Roosen et al., 2012; Linardon et al., 2017; Winkens et al., 2018), and increases in well-being (Khan and Zadeh, 2014).

Two modes of processing an experience can be differentiated: the experiential mode, characterized by direct experience of moment to moment phenomena, and a goal-directed analytical mode, characterized by judgmental or evaluative thinking (Williams, 2008). In a previous study, we asked individuals with an ED diagnosis to peel and eat an orange slice (Soler et al., 2013). Participants had to rate their level of direct engagement with the experience. Individuals who reported lower levels of direct engagement presented more severe indexes of psychopathology. Using the same task (Elices et al., 2017), compared to healthy controls (HCs), an ED group presented lower levels of direct engagement. Despite these interesting findings, the samples used in these previous studies were not large enough to discriminate between different types of eating pathologies, and individuals with OB were not tested.

Therefore, the aim of this study is to extend previous research by exploring the level of direct experience while eating in five different weight conditions. The sample contains women diagnosed with AN, BN, EDNOS, or OB, as well as individuals with no eating-related pathology (HC). No males were recruited within this research because women exhibit different patterns of eating styles (Opwis et al., 2017), with a lower presence of ED reported in men (Sweeting et al., 2015). Between-group differences in dispositional mindfulness will be explored.

## Method

### Participants and Procedure

A total sample of 143 women were recruited. The ED group [AN (*n* = 32), BN (*n* = 15), and EDNOS (*n* = 24)] was recruited in three Spanish hospitals (Hospital Sant Pau i la Santa Creu, Hospital Provincial de Castelló, and PREVI). Participants were invited by their psychologist and psychiatrist to participate in an experiment about eating. The OB group comprised 36 patients (Hospital Rector Peset), who were waiting for bariatric surgery. Participants were invited to participate by their hospital psychologist. HCs (*n* = 36) were recruited from advertisements placed around hospitals and universities. The age average was 31.1 (12.9), and for each group, the BMI was: AN (17.69), BN (24.62), EDNOS (25.83), OB (38.18), and HC (21.18) (see **Table 1**). This study was carried out in accordance with the recommendations of American Psychological Association. The study was approved by the University Jaume I (Spain) ethics committee, participation was voluntary, and signing an informed consent was required. The clinical sample was diagnosed in their own healthcare centers by experienced mental health professionals using the Structured Clinical interview for DSM-IV (SCID-I; First et al., 1999).

**Table 1:** Demographics of the sample by groups.

Demographics	AN (*n* = 32)	BN (*n* = 15)	EDNOS (*n* = 24)	OB (*n* = 36)	HC (*n* = 36)	Test statistics	
						*F*	*p*
Age				
Mean (*SD*)	22.6 (9.82)	29 (9)	32 (11.84)	24.4 (2.87)	41.60 (11.35)	22.114	<0.001
Range	14–46	15–39	17–53	21–35	24–60		
BMI Mean (*SD*)	17.69 (1.15)	24.62 (6.52)	25.83 (6.30)	38.18 (8.42)	21.18 (2.30)	69.281	<0.001
	
						*X*^2^	*p*

Educational level (%)						57.880	<0.001
No formal education	3.1%	–	–	–	–		
Primary	3.1%	–	19.0%	–	42.9%		
Secondary	53.1%	20.0%	28.6%	12.5%	35.7%		
Tertiary	9.4%	26.7%	19.0%	3.1%	–		
University	31.3%	53.3%	33.3%	84.4%	21.4%		
Marital status						58.839	<0.001
Single	62.5%	60.0%	70.8%	65.7%	–		
Live-in partner	28.1%	13.3%	–	22.9%	14.3%		
Married	6.3%	20%	4.2%	8.6%	71.4%		
Divorced	3.1%	6.7%	12.5%	2.9%	7.1%		
Widow	–	–	–	–	7.1%		


*SD, standard deviation; AN, anorexia nervosa; BN, bulimia nervosa; EDNOS, EDs not otherwise specified; OB, obesity; HCs, healthy controls; BMI, body mass index.*

Participants completed a dispositional mindfulness measure and were invited to peel an orange and eat a slice (Soler et al., 2013; Elices et al., 2017). Then, they were asked to write 10 thoughts about the task. Instructions were given to code each thought into two different categories “direct experience” (i.e., mindful mode) or “thinking about” (i.e., analytical mode). External researchers also coded the thoughts, and when disagreement arose, a group of experts decided what category fitted best. A direct experience index (DEI) was obtained by dividing the number of times a participant classified an experience as a “direct experience” (the numerator) by the total number of all observations. After the task, participants rated their level of anxiety.

### Measures

The five Facet Mindfulness Questionnaire (FFMQ; Baer et al., 2006; Cebolla et al., 2012; Aguado et al., 2015), is a 39-item questionnaire, measuring five different facets of mindfulness: Observing; Describing, Acting with awareness, Non-judging inner experience, and Non-reactivity to inner experience. Internal consistency of the FFMQ subscales for the current sample was good to excellent, [i.e., Cronbach’s alphas ranging from 0.724 (Observe) to 0.939 (Non-judging inner experience)].

Anxiety levels during the task were obtained through a visual analog scale ranging from 1 (not anxious at all) to 10 (very anxious).

### Data Analyses

Sociodemographic characteristics of the sample were analyzed using the chi-square test or univariate ANOVA as appropriate. Multivariate analyses of variance including age as covariate (MANCOVA) were performed to explore between-group differences in FFMQ. A univariate analysis of covariance with age as covariate (ANCOVA) was used to explore between-group differences in the levels of the DEI and anxiety. A partial Pearson’s correlation analysis (controlling for age) was performed to test the associations between the DEI and FFMQ and anxiety levels. Analyses were performed using IBM SPSS statistics for Windows (Version 23) (IBM Corp, 2015).

## Results

Groups differed significantly on all the sociodemographic data (**Table 1**). For BMI, results showed significantly higher values in the OB group than in the other groups (AN, BN, EDNOS, and HC) (all p’s *p* < 0.001). The AN group reported significantly lower BMI than the BN (*p* < 0.01) and EDNOS (*p* < 0.001) groups, whereas in the EDNOS group, the BMI was lower than in the HC group (*p* < 0.05).

There were statistically significant differences between the groups in the level of DEI (**Table 2**), with a large effect size. The *post hoc* Bonferroni analysis showed that the OB group scored higher than the AN (*p* < 0.001) and BN (*p* < 0.001) groups, with a large effect size. The MANCOVA test showed significant differences (*F* = 3.582; *p* < 0.001), except on the Observe facet. HC scored higher than AN (*p* < 0.001) on their describing skills, with a large effect size. On non-judging, HC scored higher than AN (*p* < 0.001) and BN (*p* < 0.001), showing the highest effect size. In the non-reactivity facet, HC score higher than AN (*p* = 0.001) and BN (*p* < 0.01), however, BN showed a larger size effect. After doing the task, the AN group reported significantly higher anxiety levels than HC (*p* < 0.05) but with a medium effect size, and the EDNOS group reported more anxiety than HC (*p* < 0.001) and OB (*p* < 0.001). The correlation analysis showed that the DEI score correlated with the Anxiety levels (*r* = -0.22; *p* < 0.05). Regarding groups, a positive correlation was found between DEI and Observe (*r* = 0.490; *p* < 0.05) and Non reactivity (*r* = 0.529; *p* < 0.05) in the EDNOS group. DEI also correlated with Awareness (*r* = 0.376; *p* < 0.05) and Non-judging inner experience (*r* = 0.401; *p* < 0.05).

**Table 2:** Between-group differences (ANOVAs) in direct experience, anxiety levels, and FFMQ.

Dependent variable	AN	BN	EDNOS	OB	HC	ANOVA	Significant *post hoc* comparisons	Cohen’s *d* [95% IC]
	*M* (*SD*)	*M* (*SD*)	*M* (*SD*)	*M* (*SD*)	*M* (*SD*)			
Direct experience index	(*n* = 32)	(*n* = 15)	(*n* = 24)	(*n* = 36)	(*n* = 36)			
	39.71 (32.13)	34.32 (31.38)	48.82 (33.48)	68.78 (23.51)	57.31 (25.61)	*F* = 5.794 *p* < 0.001	AN < OB^∗∗∗^ BN < OB^∗∗∗^	-1.03 [-1.55, -0.51] -1.33 [-2.07, -0.60]
FFMQ	(*n* = 30)	(*n* = 11)	(*n* = 18)	(*n* = 32)	(*n* = 34)			
Observing	26.00 (4.95)	27.00 (6.36)	27.61 (6.05)	22.06 (7.58)	24.88 (5.37)	*F* = 1.878 p = 0.119		
Describing	22.03 (6.96)	27.18 (8.12)	25.39 (7.75)	23.62 (7.83)	30.25 (4.22)	*F* = 5.434 *p* < 0.001	HC > AN^∗∗∗^	-1.42 [-1.98, -0.86]
Acting with awareness	25.07 (5.71)	25.00 (4.43)	23.39 (7.49)	22.12 (7.64)	28.94 (5.87)	*F* = 2.360 *p* < 0.05		
Non-judging	21.37 (6.28)	20.00 (6.15)	18.22 (8.24)	21.97 (8.64)	30.34 (5.80)	*F* = 10.520 *p* < 0.001	HC > AN^∗∗∗^ HC > BN^∗∗^ HC > EDNOS^∗∗∗^	1.47 [0.91, 2.03] 1.72 [0.92, 2.50] 1.76 [1.09, 2.44]
Non-reactivity	17.60 (4.60)	16.63 (3.41)	18.83 (4.62)	18.24 (5.69)	22.41 (3.93)	*F* = 5.353 *p* < 0.01	HC > AN^∗∗^ HC > BN^∗∗^	1.11 [0.58, 1.64] 1.49 [0.73, 2.23]
Anxiety	(*n* = 23)	(*n* = 12)	(*n* = 24)	(*n* = 36)	(*n* = 36)			
	3.37 (2.59)	3.33 (2.70)	5.10 (2.87)	2.74 (2.74)	1.94 (2.18)	*F* = 10.208 *p* < 0.001	AN > HC^∗^ EDNOS > HC^∗∗∗^ EDNOS > OB^∗∗∗^	0.59 [0.08, 1.10] 1.13 [0.57, 2.05] 0.84 [0.14, 1.54]


*AN, anorexia nervosa; BN, bulimia nervosa; EDNOS, EDs not otherwise specified; OB, obesity; HCs, healthy controls. ^∗^p < 0.05; ^∗∗^p < 0.01; ^∗∗∗^p < 0.00.*

## Discussion

The current findings partially confirmed the previous literature (Soler et al., 2013; Elices et al., 2017), as the more severe ED had lower access to direct experience while eating. Unexpectedly, the OB group did not respond in the same way as the other clinical groups, presenting similar results to HC. Consistent with previous research, in the ED groups the experience of eating generated higher anxiety. Furthermore, a negative correlation was found between the DEI and anxiety scores. Interestingly, Soler et al.’s (2013) study, also using an ED sample (combining AN, BN, and EDNOS), revealed that the DEI was heavily influenced by anxiety levels experienced during the task, followed by ED severity and one facet of the FFMQ. The interrelation between anxiety and direct experience could be interpreted according to the Mindfulness-Based Cognitive Therapy view, in which analytical thinking is considered a feature of the doing mode, a mindless state (Williams, 2008, 2010). When a discrepancy is detected between a given reality (e.g., actual weight) and what is expected or desired (e.g., ideal weight), this mode is activated, rumination starts, and negative emotions (i.e., anxiety) arise and last until the discrepancy is resolved (Segal et al., 2002).

We found comparable results for OB and HC on DEI, which could be interpreted as an apparently better “mindful” contact with the eating experience, compared to the ED groups. Furthermore, the results on the FFMQ response in the OB group was surprising because previous studies found that mindfulness facets were diminished in this group (Lavender et al., 2011). Thus, the question arises: why is the DEI of OB individuals comparable to that of HC? Williams (2008) differentiates indirect (conceptual) experience versus direct (non-conceptual) experience as one of the pairs of characteristics associated with the mindless versus mindfulness modes. However, there are five additional pairs: (1) Striving versus Non-striving, (2) Avoidance versus Approach, (3) considering thoughts to be “real” versus thoughts as mental events, (4) living in the past and future versus living in the present moment, and, (5) automatic versus intentional. The orange experiment is intended to capture the first pair -analytical versus direct experience-, but not the others. Thus, an increase in direct experience alone may not be representative of a complete mindfulness experience. Moreover, “increased” experience could be observed in clinical conditions (i.e., hypochondriac) without implying a mindful stance.

Another aspect that could explain this result is the concept of savoring, “the process through which people attend to positive experiences and engage in thoughts and behaviors that regulate positive feelings that arise from these experiences” (Bryant, 1989). Savoring has been suggested as a mechanism through which people derive happiness from positive events (Jose et al., 2012). The DEI could also measure savoring; peeling an orange and eating it can be a delightful experience for OB but not for ED. In fact, in the OB, the DEI score does not correlate with the FFMQ. Although mindfulness and savoring have been related (Bryant and Smith, 2015), they also differ because savoring does not imply other aspects of mindfulness (i.e., body awareness, acceptance of inner experience, or decentering). Further research is needed to establish whether the orange task is a measure of savoring, mindful eating, or both, and whether this task can be used as a change measure after a mindful eating program.

The use of mindful eating training in ED is increasing, similar to the situation in OB. This study confirms that this type of training could be helpful to decrease the anxiety response to food cues and confront the fear of eating. However, more research is needed when using it in an OB group because mindful eating may be useful only as a savoring tool, and not to modify eating behaviors.

This study has several limitations, including the small sample size, the lack of a male sample, the heterogeneity of samples in terms of age, and other sociodemographic variables, as well as the lack of a specific questionnaire for measuring mindful eating. However, the orange task seems to overcome some of the limitations associated with self-report questionnaires, which present weak psychometric properties and have to be used with caution (Park et al., 2013). In this regard, there is a demand for laboratory measures of mindfulness (Visted et al., 2015; Baer, 2016). Few proposals have shown excellent and promising results (Levinson et al., 2014), and they are a critical step in advancing the field.

## Author Contributions

JS and AC conceptualization and supervision. JS, AC, DC, and ME formal analyses. JS, AC, CM-B, DM-R, XA, CC, MJ, GL, and VG methodology. DC, DM-R, JS, AC, and ME writing and original draft. JS, AC, CB, and RB writing, review, and editing.

## Conflict of Interest Statement

The authors declare that the research was conducted in the absence of any commercial or financial relationships that could be construed as a potential conflict of interest. The reviewer RK and handling Editor declared their shared affiliation.
